# Supplementary material for: HCV- and HBV-mediated liver cancer converge on similar transcriptomic landscapes and immune profiles
Source: bioRxiv. 2024 Jul 3:2024.07.01.601493. Preprint. [Version 1] doi: 10.1101/2024.07.01.601493 (PMC11244919; doi:10.1101/2024.07.01.601493)
Supplement: Supplement 1 [file NIHPP2024.07.01.601493v1-supplement-1.pdf]

bioRxiv preprint doi: https://doi.org/10.1101/2024.07.01.601493; this version posted July 3, 2024. The copyright holder for this preprint (which was not certified by peer review) is the author/funder. This article is a US Government work. It is not subject to copyright under 17 USC 105 and is also made available for use under a CC0 license.

Supporting Information Captions

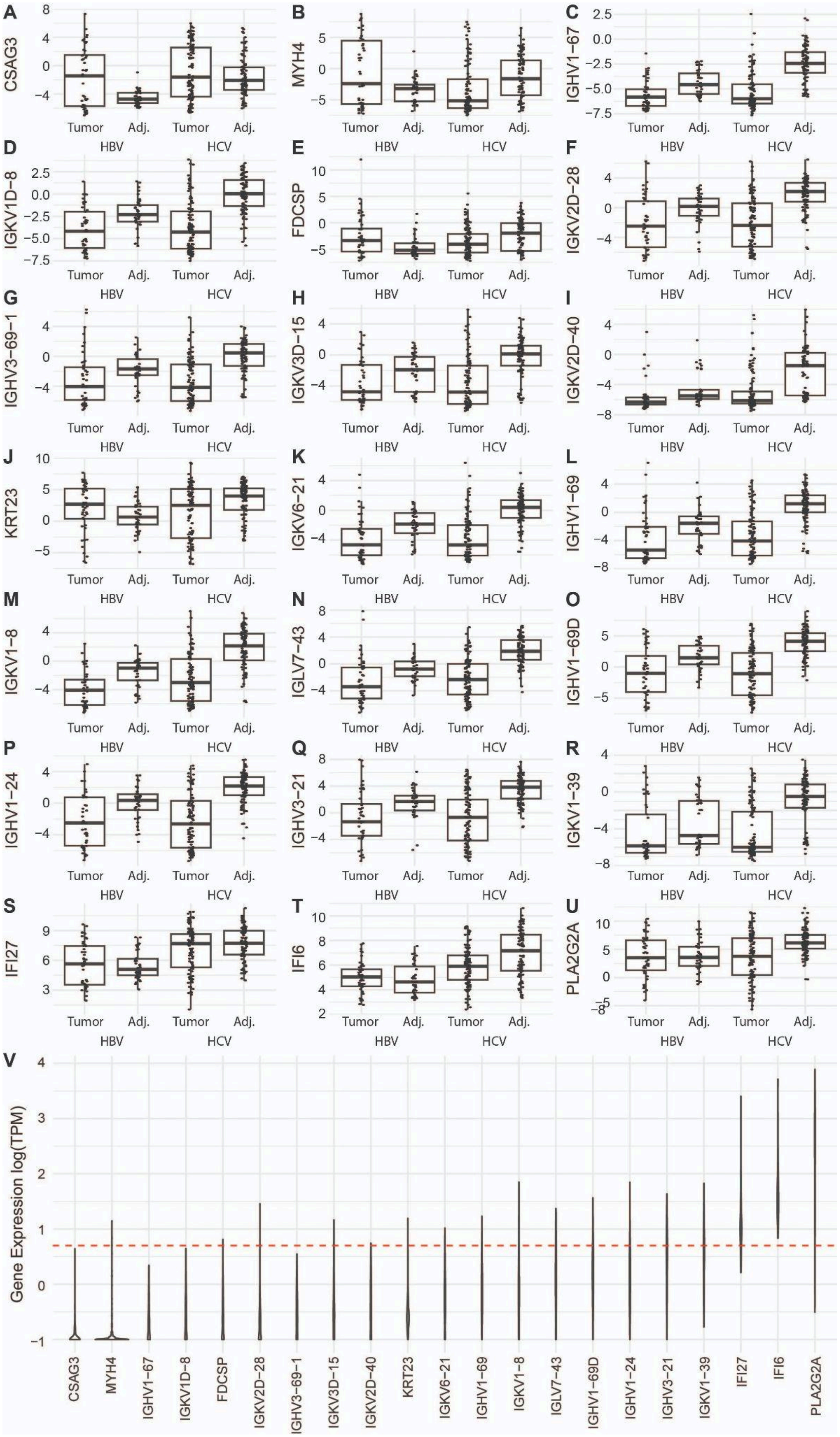

bioRxiv preprint doi: https://doi.org/10.1101/2024.07.01.601493; this version posted July 3, 2024. The copyright holder for this preprint (which was not certified by peer review) is the author/funder. This article is a US Government work. It is not subject to copyright under 17 USC 105 and is also made available for use under a CC0 license.

Supplementary Figure 1: Expression of top differentially expressed genes from HBV tumor-adjacent to HCV tumor-adjacent tissue. (A-U) Boxplots demonstrating the *voom* normalized expression of each gene upregulated in HCV vs. HBV tumor-adjacent samples across tumor and tumor-adjacent, HBV and HCV samples. **(V)** Violin plot of the expression of genes upregulated in HCV tumor-adjacent tissue compared to HBV tumor-adjacent tissue in healthy liver tissue from the GTEx dataset. Expression in GTEx is normalized as transcripts per million (TPM) and is displayed on a log base 10 scale. The horizontal red dashed line corresponds to 1 TPM of gene expression.

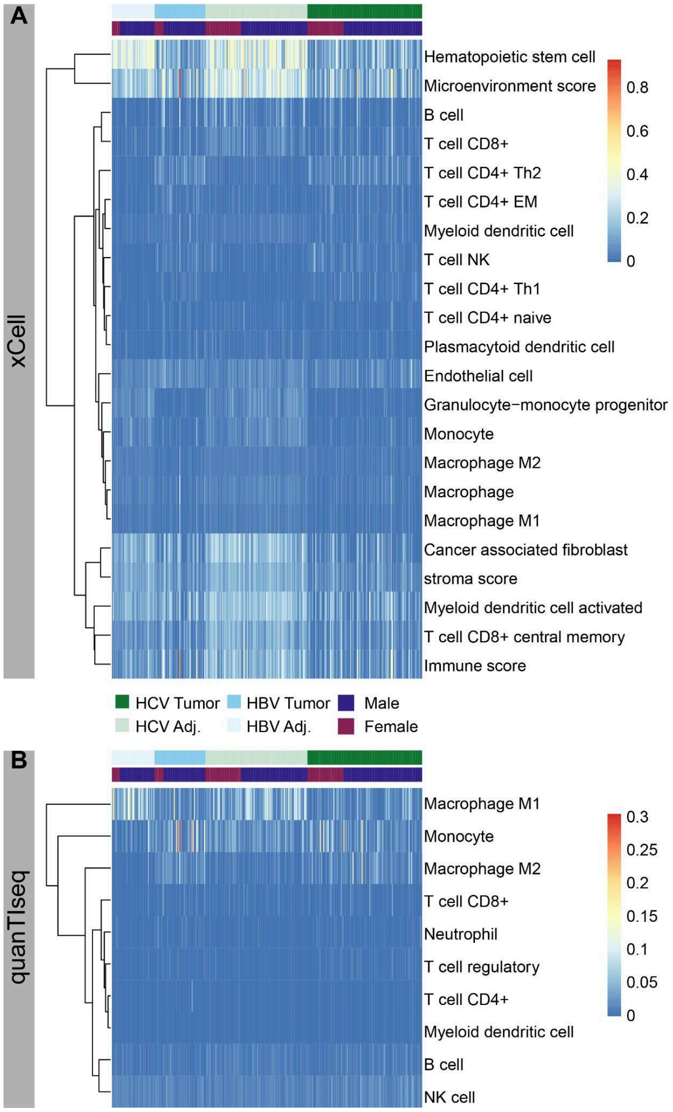

Supplemental Figure 2: Full xCell and quanTIseq results across HBV and HCV samples. Heatmap of all immune cells identified with **(A)** xCell and **(B)** quanTIseq. Each column represents a single sample and the annotation bars across the top separate HCV tumor, HCV adjacent, HBV tumor, HBV adjacent, and male and female samples. Ajd.; adjacent.

bioRxiv preprint doi: https://doi.org/10.1101/2024.07.01.601493; this version posted July 3, 2024. The copyright holder for this preprint (which was not certified by peer review) is the author/funder. This article is a US Government work. It is not subject to copyright under 17 USC 105 and is also made available for use under a CC0 license.

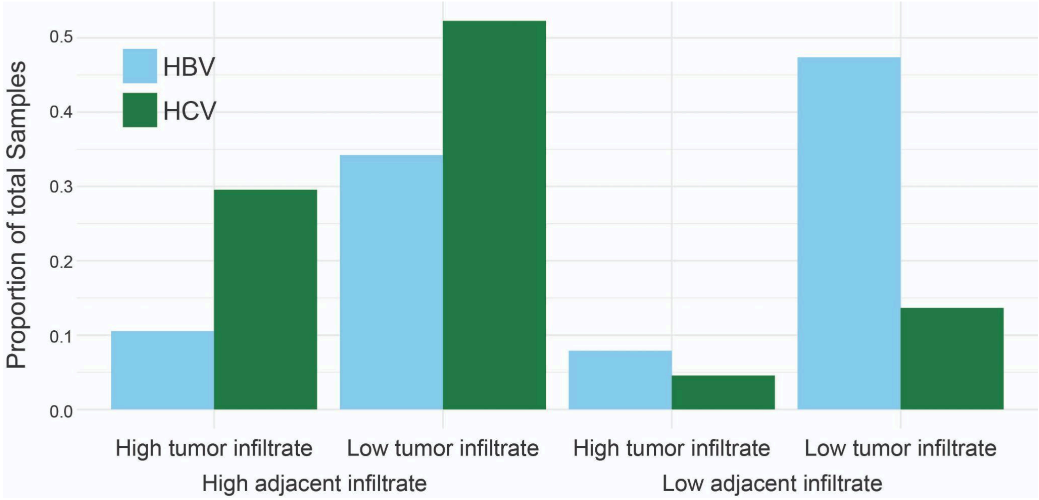

Supplemental Figure 3: Comparison of infiltrate in tumor and adjacent matched samples. Comparison of the percent of HBV and HCV samples that fall into one of four categories of immune infiltration, high adjacent and tumor infiltration, high adjacent and low tumor infiltration, low adjacent and high tumor infiltration, and low adjacent and low tumor infiltration. High infiltration is defined as any infiltration above the median of all samples and low is defined as any infiltration below the median of all samples

bioRxiv preprint doi: https://doi.org/10.1101/2024.07.01.601493; this version posted July 3, 2024. The copyright holder for this preprint (which was not certified by peer review) is the author/funder. This article is a US Government work. It is not subject to copyright under 17 USC 105 and is also made available for use under a CC0 license.

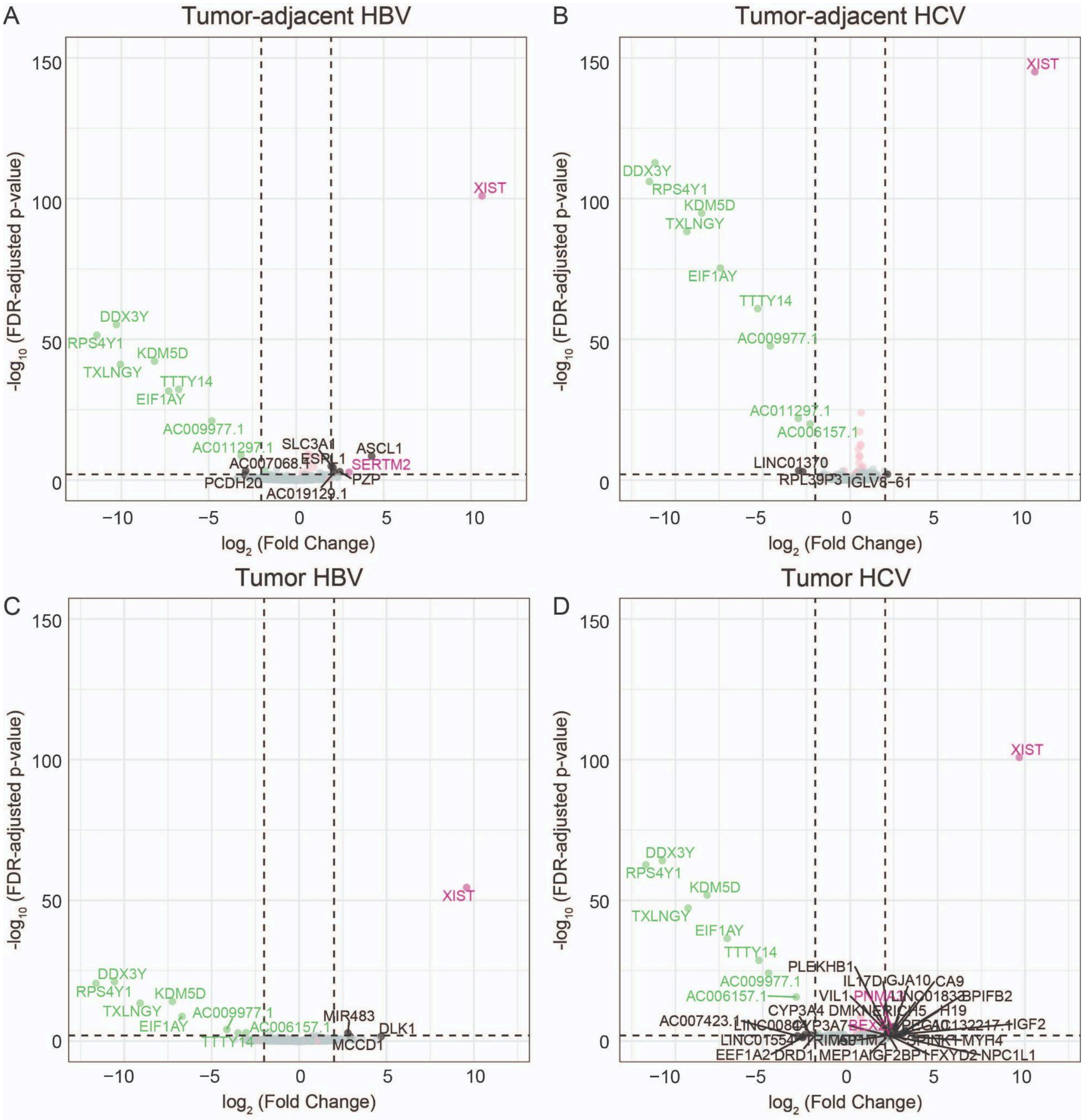

Supplementary Figure 4: A-D) Volcano plots of differentially expressed genes from **(A)** male HBV tumor-adjacent:female HBV tumor-adjacent samples, **(B)** male HCV tumor-adjacent:female HCV tumor-adjacent samples, **(C)** male HBV tumor:female HBV tumor samples, and **(D)** male HCV tumor:female HCV tumor samples. X-linked genes are indicated in pink, Y-linked in green, and autosomal in black.

bioRxiv preprint doi: https://doi.org/10.1101/2024.07.01.601493; this version posted July 3, 2024. The copyright holder for this preprint (which was not certified by peer review) is the author/funder. This article is a US Government work. It is not subject to copyright under 17 USC 105 and is also made available for use under a CC0 license.

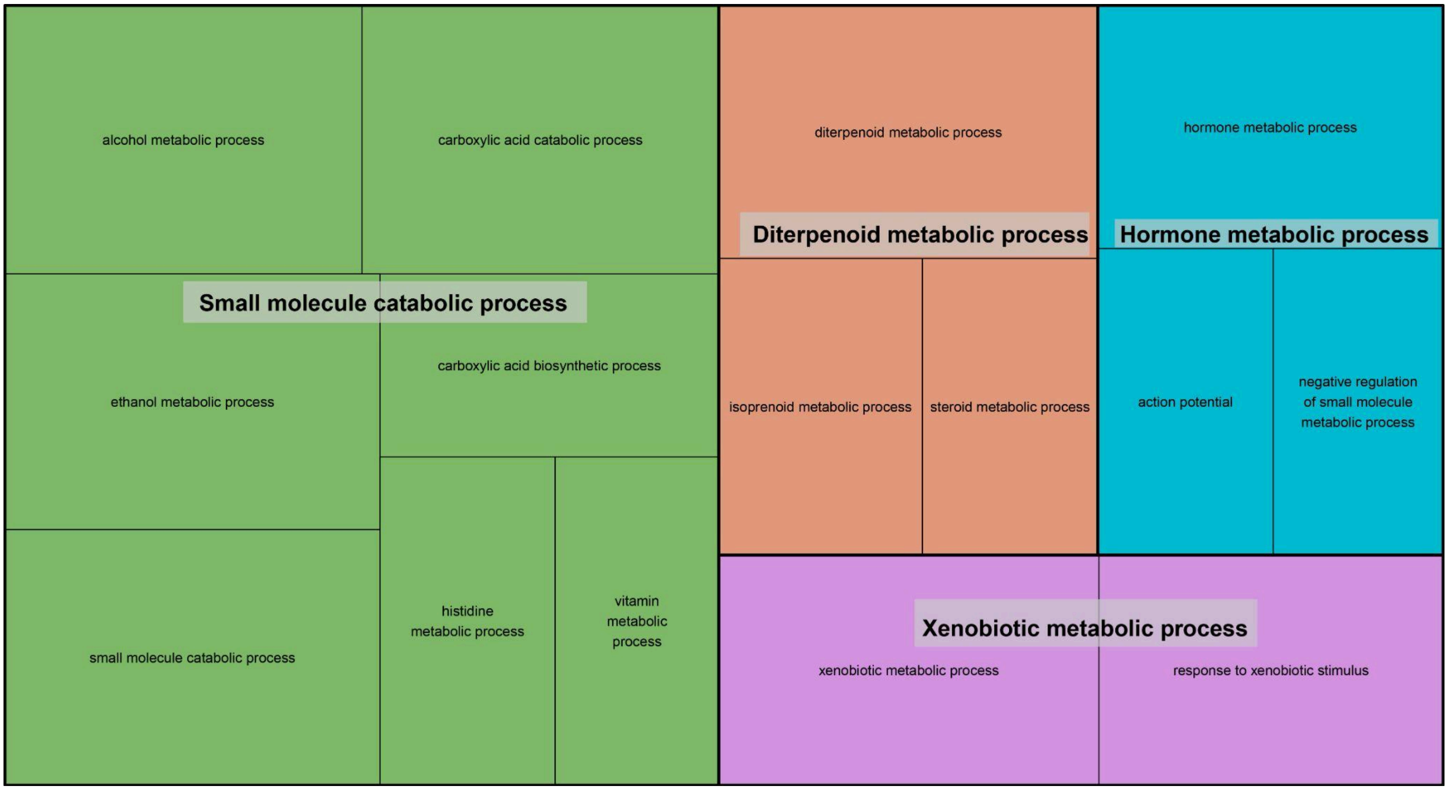

Supplementary Figure 5: Pathways enriched in male HCV tumor:female HCV tumor comparison. Treemap visualization of GO enrichment analysis from all differentially expressed genes in the male HCV tumor:female HCV tumor comparison. The sizes of the boxes reflect the magnitude of the false-discovery adjusted p-value for the GO enrichment term.

bioRxiv preprint doi: https://doi.org/10.1101/2024.07.01.601493; this version posted July 3, 2024. The copyright holder for this preprint (which was not certified by peer review) is the author/funder. This article is a US Government work. It is not subject to copyright under 17 USC 105 and is also made available for use under a CC0 license.

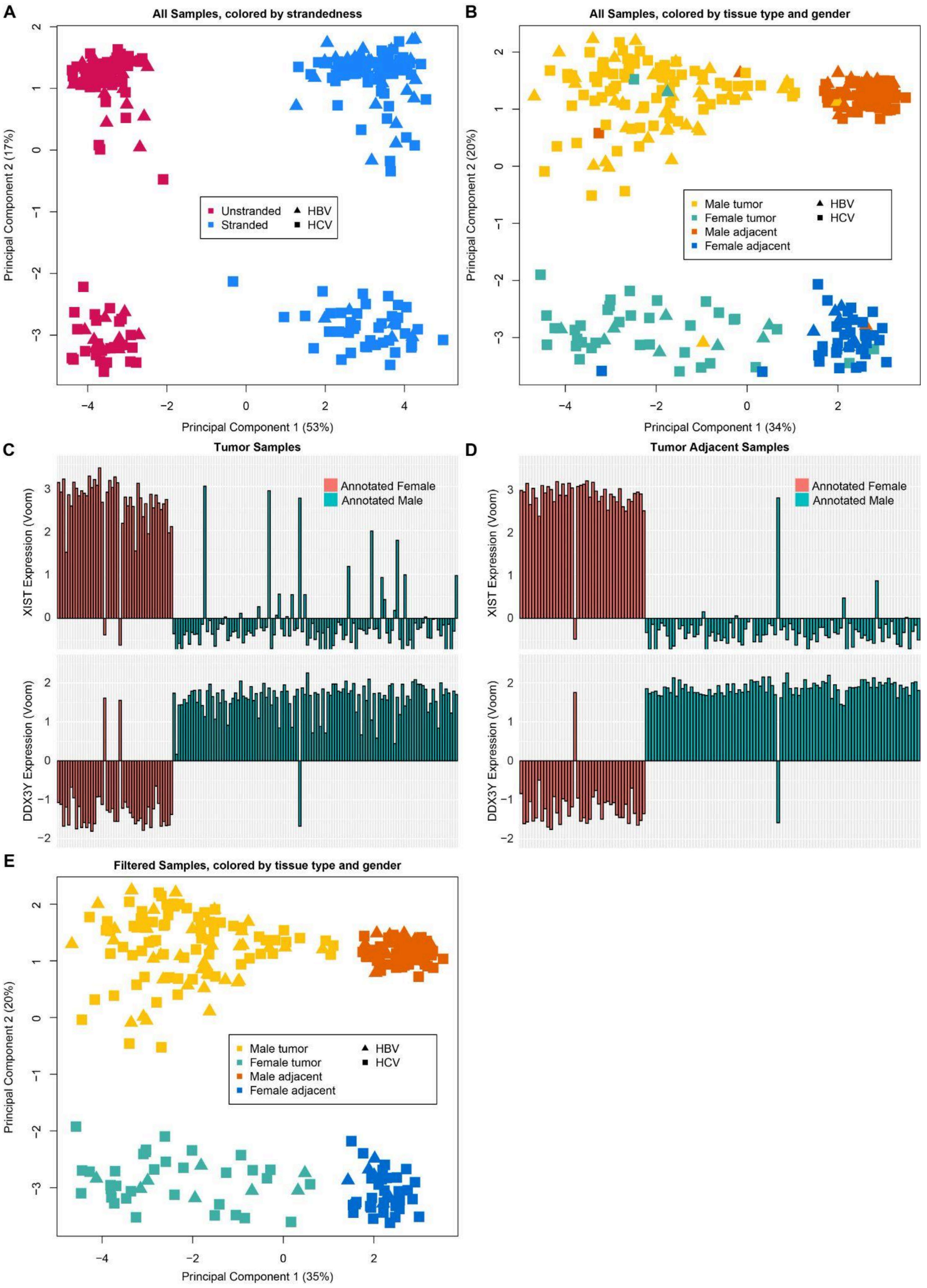

Supplementary Figure 5: Quality control of all samples on tumor type and sex. MDS plot on the top 25 most variable genes, colored by **(A)** library type and **(B)** tumor status and sex. Plot of expression of XIST and DDX3Y across all **(C)** tumor samples and **(D)** tumor-adjacent samples. RK106, RK135, and RK105 were removed from subsequent analyses due to likely mislabeled sex supported by the MDS plots and expression of XIST and DDX3Y. RK066, RK113, and RK116 were removed due to the proximity of the paired sample suggesting that there may be cross-contamination of the samples. Finally, RK179 and RK065 had tumor and tumor-adjacent samples that were in opposite clusters on the MDS plot, therefore, these samples were relabeled to be consistent with their observed clusters. **(E)** MDS plot on the top 25 most variable genes colored by tumor status and sex.
